# Post‐traumatic stress symptoms in long‐term disease‐free cancer survivors and their family caregivers

**DOI:** 10.1002/cam4.3961

**Published:** 2021-06-01

**Authors:** Silvia De Padova, Luigi Grassi, Alessandro Vagheggini, Martino Belvederi Murri, Federica Folesani, Lorena Rossi, Alberto Farolfi, Tatiana Bertelli, Alessandro Passardi, Alejandra Berardi, Ugo De Giorgi

**Affiliations:** ^1^ Psycho‐Oncology Unit Istituto Scientifico Romagnolo per lo Studio e la Cura dei Tumori (IRST) IRCCS Meldola Italy; ^2^ Department of Biomedical and Specialty Surgical Sciences University of Ferrara and University Hospital Psychiatry Unit Integrated Department of Mental Health S. Anna University Hospital and Health Authorities Anna University Hospital Ferrara Italy; ^3^ Unit of Biostatistics and Clinical Trials Istituto Scientifico Romagnolo per lo Studio e la Cura dei Tumori (IRST) IRCCS Meldola Italy; ^4^ Department of Medical Oncology Istituto Scientifico Romagnolo per lo Studio e la Cura dei Tumori (IRST) IRCCS Meldola Italy

**Keywords:** cancer survivors, family caregivers, posttraumatic stress symptoms, psychological distress

## Abstract

**Background:**

The experience of cancer is highly stressful and potentially traumatic. We assessed the presence of Post‐Traumatic Stress Symptoms (PTSS) in long‐term cancer survivors and their caregivers, while examining the association between PTSS and clinical, demographic and psychological variables in the long term.

**Methods:**

In this cross‐sectional study 212 survivor‐family caregiver dyads completed measures of post‐traumatic stress symptoms (PTSS) (Impact of Event Scale), depression and anxiety (Hospital Anxiety Depression Scale). Coping strategies, fatigue, cognitive decline, stressful life events and psychopathological history were also assessed among survivors. Data were analyzed using mixed models, accounting both for individual and dyadic effects.

**Results:**

Cancer survivors and their caregivers were assessed after a mean of 6 years after treatment. Twenty per cent of survivors and 35.5% of caregivers had possible posttraumatic stress disorder (PTSD), while 23 patients (11.0%) and 33 caregivers (15.6%) had probable PTSD. Among cancer patients, the severity of post‐traumatic symptoms was associated with an anxious coping style, previous psychopathology and depression (*p* < 0.001), whereas among caregivers it was associated with depression and having a closer relationship with patients (*p* < 0.001). Patients’ depression was associated with caregivers’ intrusion symptoms.

**Conclusions:**

High levels of cancer‐related PTSS were still present several years after treatment in both survivors and caregivers. Psychopathology may derive from complex interactions among coping, previous disorders and between‐person dynamics.

## Graphical abstract

High levels of cancer‐related PTSS are still present several years after treatment in both testixular cancer survivors and caregivers. Psychopathology may derive from complex interactions between coping, previous disorders and between‐person dynamics.

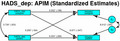

## 1 INTRODUCTION

Cancer brings a significant psychological burden, ranging from adjustment difficulties to anxiety, depression or even Post‐Traumatic Stress Disorder (PTSD). The psychological consequences of cancer affect both patients and their caregivers, who also frequently develop clinically meaningful disorders. Disorders may derive from witnessing sufferance in a loved one as well as enduring multiple lifestyle and psychosocial upheavals.^1^, ^2^, ^3^, ^4^ It is increasingly recognized that such phenomena are not limited to the immediate period after cancer diagnosis, but may outlast the remission phase even for years. Cancer, in fact, is becoming a chronic disorder, with the rates of long‐term survivors increasing worldwide. Despite these premises, most research on cancer‐related psychopathology is limited to the initial stages of cancer management,^5^, ^6^ and has generally overlooked the reciprocal interactions between patients’ and caregivers’ symptoms.^7^, ^8^, ^9^ Few studies examined trauma‐related psychopathology among cancer survivors. Mehnert et al. reported that 12% of 1,083 long‐term survivors of breast cancer were diagnosed with PTSD.^10^ PTSD significantly reduced the quality of life of patients and caused significant distress even in their offspring.^11^ Shelby et al. observed that 10%–20% of cancer survivors presented with sub‐syndromal levels of PTSD also termed Post‐Traumatic Stress Symptoms (PTSS), which were also sufficient to determine a significant reduction of the quality of life.^12^ Among cancer survivors, PTSD or PTSS may display a prevalence ranging from 7.3% to 13.8%, according to a standard operational scoring system or diagnostic criteria, respectively.^13^, ^14^, ^15^ Data on PTSD/PTSS are particularly scarce among caregivers of cancer patients (usually spouses, parents or siblings). Relatives of cancer patients, in fact, are elective recipients of distressing, or even traumatic experiences of a life‐threatening disease. They are most frequently endowed with the caregiver role during and after treatment, and are chronically exposed to the uncertainty of the evolution of the illness, as well as to the psychosocial and economic impact of cancer and day‐to‐day management. Most studies examined the experience of caregivers in the acute phase of treatment or in the first months after diagnosis. Surprisingly, they suggested that caregivers might suffer similar or even higher rates of PTSD than patients.^16^, ^17^, ^18^ To date, the majority of research on long‐term survivors has focused on family members and survivors of paediatric cancers.^19^, ^20^, ^21^ Here, the prevalence of clinically significant PTSS ranged from 4.4% to 78% and those of PTSD from 0% to 34.8%.^22^ Knowledge on relatives of adult cancer survivors is even more scarce: to our knowledge, only a recent study examined 93 long‐term survivors of head and neck cancer and their partners, reporting that 15.4% of patients and 12.8% of partners could be diagnosed with PTSD, while an additional 33.3% of patients and 25.7% of partners displayed significant PTSS. There were no significant differences in the severity of PTSS between patients and their partners.^23^ Despite the lack of available studies, it can be hypothesized that both PTSS and PTSD are a significant cause of distress for cancer survivors and their caregivers, and may have an impact that is comparable to other symptoms, such as depression.^24^ Given these premises, the purpose of this study was to investigate the prevalence, correlates of post‐traumatic symptoms in a sample of long‐term cancer survivors. Considering the importance of the relationship between patients and caregivers, we also aimed at examining patient‐caregiver dyadic symptom interactions, as they may highlight specific, individual contributions. In particular, our aims were: (i) to estimate the prevalence of post‐traumatic symptoms among survivors and caregivers; (ii) to identify their psychological, clinical and sociodemographic correlates, and (iii) to examine the relationship between the facets of post‐traumatic symptoms (intrusion, avoidance and anxiety) while accounting for their interdependence in the members of the dyad.^25^ Considering prior evidence, we hypothesized that survivors would display a high prevalence of post‐traumatic symptoms, and they would display significant associations across the members of the dyad.

## 2 MATERIALS AND METHODS

### 2.1 Participants and procedure

This study focused on a sample of cancer survivors (n = 212) and their family caregivers (n = 212). Between 2011 and 2017, 300 potential participants included in the database of the “free of cancer” program of our Institute (Istituto Scientifico Romagnolo per lo Studio e la Cura dei Tumori IRST IRCCS) were contacted during the follow‐up visit at our institute or by phone/email and were invited to participate in the study with their family caregivers. Of these, 279 (93%) patients agreed to participate, but a family caregiver was identified and accepted the invitation in only 212 (76%) cases. All 212 patient‐relative dyads took part in a 1‐hour face‐to face interview conducted by the same clinical psychologist (S.D.), during which medical and psychosocial data were collected and a series of questionnaires administered.

Eligibility criteria for LCS were age >18 years, continuous disease‐free status after treatment for a prior cancer, a minimum of 5 years’ follow‐up after treatment completion and a good understanding of the Italian language. Patients were required to give written informed consent. Family caregivers were selected by asking each survivor to identify a relative who had been most involved in the disease experience and follow‐up. Inclusion criteria for family caregivers were nomination by the cancer survivor, age >18 years, and Italian‐speaking. All provided written informed consent. Exclusion criteria for both groups were poor literacy, cognitive deficit or psychiatric disorder.

Given that there are various definitions of the concept of cancer survivorship, we used a distinct clinical meaning of the term ‘survivor’ referring to individuals who have had a life‐threatening disease, but have remained disease‐free for a minimum of 5 years.^26^, ^27^ Patients and caregivers who agreed to participate in the study underwent informed consent procedures in accordance with Institutional guidelines. They were then administered a one‐time survey using standardized questionnaire measures. Survivors and caregivers were asked to complete the survey separately and not to consult each other with regard to their answers. The participants were asked multiple‐choice questions on psychopathological history: disorder type (anxiety disorder, depression, sleep disturbances, etc.), period of life in which it arose, duration, possible pharmacological and/or psychotherapeutic intervention and stressful life events. The study was approved by IRST’s Ethics Committee and was conducted in accordance with the ethical standards laid down in the 1964 Declaration of Helsinki. A signed informed consent was provided by all participants.

### 2.2 Measures

A set of psychometric tools was administered to each patient‐relative dyad to assess PTSS and other symptoms, including anxiety and depression. Patients and relatives were assessed with the Impact of Events Scale (IES) and the Hospital Anxiety and Depression Scale (HADS).

The IES^28^ is a 15‐item tool rating the severity of PTSS on a 6‐point Likert scale (0 = not at all to 5 = often). The scale consists of two subscales, intrusion (7 items, score range 0–35) and avoidance (8 items, score range 0–40). It has been widely used in cancer settings,^29^, ^30^ and validated in Italian.^31^ The IES‐total score (range 0–75) was used as an index of PTSS severity: ≥35 (severe), 26–34 (moderate) and <26 (mild).

The HADS^32^ was used to assess the severity of anxiety and depression symptoms. The HADS is a 14‐item questionnaire, validated in Italian,^33^ which is frequently employed in both cancer^34^ and general population^35^ settings. It consists of two subscales, depression (HADS‐D, 7 items) and anxiety (HADS‐A, 7 items). Recently, it was noted the items of the anxiety subscale largely assess symptoms related to restlessness, panic and fright, thus we used its score as an index of hyperarousal.^36^ Each item is scored on a four‐point scale (from 0 = not present, to 3 = considerable), with scores for each subscale ranging from 0 to 21. A cut‐off score ≥8 for either subscale has been commonly employed to detect clinically significant anxiety and depression symptoms in cancer patients.^35^

To estimate the prevalence of PTSD we relied on multiple criteria. Used as a screening tool, an IES total score equal or greater than 35 was shown to optimize sensitivity (0.89) and specificity (0.94) for a diagnosis of PTSD assessed with DSM‐IV criteria.^37^, ^38^ However, while the IES provides a measure of avoidance and intrusion symptoms, it does not assess hyperarousal, which is required by DSM‐IV. Hence, we used this definition (IES score ≥35) to estimate the prevalence of *possible* cases of PTSD. In addition, to increase specificity we combined this criteria with the presence of hyperarousal, indexed by the HADS‐A, to estimate the prevalence of *probable* PTSD.

Adjustment to cancer was assessed with the Mini‐Mental Adjustment to Cancer (Mini‐MAC) questionnaire. The Mini‐MAC^39^ in its validated Italian version^40^ has also been used in LCS to assess patients’ cognitive and behavioural attitudes towards cancer. As already done in other studies,^41^ two subscales on a 1–4 Likert scale (1 = it definitely does not apply to me; 4 = it definitely applies to me) were used to assess maladaptive coping, namely hopelessness‐helplessness (Mini‐MAC/H, 8 items, measuring adrift and impotence, with consequent, withdrawing from social life and professional activity); and anxious preoccupation (Mini‐MAC/AP, items), indicative of disease‐related fear and perceiving disease as a worrying, uncontrolled, and threatening phenomenon.

To identify pre‐existing or current stressors that may have contributed to survivor PTSS, patients were asked a single‐item question, “Are there any other stresses in your life that you find as stressful as the experience of cancer?” (YES/NO response). Caregivers were asked to express their perceptions of the survivor's health with a single‐item question on how they would rate the health of their relative. The item was adapted from the Italian version of the Short‐Form‐36 Health Survey Questionnaire^42^ and used a five‐level rating (excellent, very good, good, fair, poor).

### 2.3 Statistical analysis

First, we reported descriptive data relative to sociodemographic, clinical and psychometric characteristics of the sample. Continuous variables were summarized as mean values and standard deviations, whereas absolute (i.e. counts) and relative (i.e. percentages) frequencies were reported for categorical variables. The total number of missing values for each variable, if present, was also shown.

Second, we compared patients and caregivers sociodemographic and clinical features, using paired t‐tests for continuous variables and Cohen's kappa test for categorical variables. We also assessed the level of feature interdependence for each shared variable using Pearson pairwise correlation for continuous variables and Cohen's kappa for categorical variables, as recommended when examining distinguishable dyads.^43^

Third, we examined the associations between symptom scores using pairwise Pearson correlations.

Fourth, we examined the associations between sociodemographic and clinical factors and clinical domains of PTSD, within the patient and caregiver groups, using three multiple linear regression models with intrusion, avoidance and anxiety scores as the dependent variables. These analyses were performed with SPSS, 23.0 version.

Fifth, we examined the association between clinical symptoms accounting for the interdependence of dyad members’ data. To this end, we run separate Actor‐Partner Interdependence Models (APIM),^44^ based on multilevel analyses, using the web‐based Shiny interactive app built on the *dyadR* package for R.^43^, ^45^ In particular, three multilevel APIMs were employed to examine the patient/caregiver distinguishable association between depression (used as the predictor) and intrusion, avoidance and anxiety (used as the dependent variables). One additional APIM was run to examine the association between intrusion and anxiety (used as predictors) and avoidance (used as the dependent variable). For all analyses, the *α* = 0.05 significance level was adopted. The detailed models’ outputs are reported in the supplement.

## 3 RESULTS

### 3.1 Sociodemographic, clinical and psychological characteristics

A sample of 212 cancer survivors and 212 family caregivers were interviewed after 5–19 years (mean 6 ± 4 years, the median time of remission = 6 years) cancer treatment had ended. Primary cancer sites were breast (35.8%), testicle (21.2%), whereas the remaining sites included colorectal, gastric, gynaecological, kidney, prostate and bladder cancer (42.9%). The most common treatment regimens were surgery alone (25.9%) and a combination of chemotherapy or hormone therapy with other treatments (42%), while 32.1% of patients underwent radiation therapy alone or in combination with other therapies. Almost all of the participants had non‐metastatic cancer (92.3%) and no disease recurrence (90%). Half of the patients (52.9%) had one or more comorbidities (Table 1).

**TABLE 1:** Socio‐demographic and clinical data of survivors and caregivers

	Survivors no. (%)	Caregivers no. (%)	Statistics
Mean age, years (SD)	59.3 (14.8)	57.5 (14.45)	*R* = 0.42; *p* < 0.001
Unknown	1	2	
Gender			Cohen K *p* < 0.001
Male	88 (41.5)	93 (43.9)	
Female	124 (58.5)	119 (56.1)	
Education			Cohen *K* *p* < 0.001
Primary school	24 (11.4)	36 (17.0)	
Middle school	81(38.4)	83 (39.2)	
High school	53 (25.1)	54 (25.5)	
University	53 (25.1)	39 (18.4)	
Unknown	1	‐	
Employment status			Cohen *K* *p* < 0.001
Currently working	79 (37.6)	84 (39.6)	
Retired	105 (50.0)	91 (42.9)	
Other	26 (12.4)	37 (17.5)	
Unknown	2	‐	
Marital status			Cohen *K* *p* < 0.001
Single	21 (10.0)	18 (8.5)	
In a relationship	168 (79.6)	180 (84.9)	
Divorced/widowed	22 (10.4)	14 (6.6)	
Unknown	1		
Children			
Yes	44 (20.9)		
No	167 (79.1)		
Unknown	1		
Family caregivers			
Parent		22 (10.5)	
Spouse/partner		141 (67.1)	
Other		47 (22.4)	
Unknown		2	
Mean time since remission, years (SD)	6.1 (2.5)		
Tumour location			
Testicle	45 (21.2)		
Breast	76 (35.8)		
Other	91 (42.9)		
Treatment			
Surgery	55 (25.9)		
CT or HT + other treatment	89 (42.0)		
RT + other treatment	68 (32.1)		
Metastasis			
Yes	16 (7.7)		
No	193 (92.3)		
Unknown	3		
Relapse^a^			
Yes	21 (10.0)		
No	190 (90.0)		
Unknown	1		
Co‐morbidities			
Yes	111 (52.9)		
No	99 (47.1)		
Unknown	2		
Caregivers’ perception of health of survivor			
Excellent	17 (8.0)		
Very good	41 (19.3)		
Good	118 (55.7)		
Acceptable	30 (14.2)		
Poor	6 (2.8)		

Abbreviations: CT, chemotherapy; HT, hormone therapy; SD, standard deviation.

^a^ Patients relapsed after the primary treatment, who achieved a further disease‐free status.

Patient history taking revealed that 89 (42%) cases had suffered from at least one psychopathological condition such as anxiety or depression for 12 months or longer before the diagnosis of cancer, while 22 (10.3%) reported psychopathological disorders lasting less than 6 months (n = 13, 6.1%) or between 6 and 12 months (n = 9, 4.2%). Among those who experienced the problems before diagnosis (n = 111, 52.3%), 43 (38.7%) were treated with psychopharmaceuticals and 25 (22.5%) underwent psychotherapy. The majority of subjects (n = 165, 79.3%) reported stressful situations at different times (before, during or after the illness).

The mean age of cancer survivors was 59 years (standard deviation [SD] = 14.9 years), while the mean age of family caregivers was 57.5 years (SD = 14.5; *p* = 0.202). Most family members were spouses or partners (67.1%), 10.5% were parents and 22.4% were siblings, children or other relatives. As expected, most sociodemographic factors were interdependent between patients and caregivers (Pearson and Cohen kappa *p *< 0.05).

### 3.2 PTSS and psychosocial variables

Paired t‐test and Pearson's correlations were used to compare the symptom severity between survivors and caregivers, and results are reported in Table 2. A significant difference as well as interdependence between patients and caregivers were observed regarding PTSS symptoms of avoidance (*t*‐test *p* = 0.001; *R* = 0.285) and intrusion (t‐test *p* < 0.001; *R* = 0.264), but not anxiety. Depression displayed a significant correlation (*R* = 0.150, *p* = 0.038) but not a significant difference between survivors and caregivers.

**TABLE 2:** Comparison of symptom severity in the sample

	Survivors		Caregivers		*p* ^b^	*R* ^a^
	Mean	SD	Mean	SD		
IES Avoidance	18.3	6.3	20.2	6.9	0.001*	0.285*
Testicle	18.4	5.6	19.8	5.6	0.290	0.205
Breast	17.7	6.4	19.2	6.4	0.081	0.335*
Other	18.3	6.7	21.3	7.1	0.021*	0.145
IES Intrusion	11.8	4.7	14.6	5.3	<0.001*	0.264*
Testicle	11.2	3.7	15.0	5.9	<0.001*	0.138
Breast	12.1	5.0	13.8	5.1	0.019*	0.303*
Other	10.9	4.7	15.0	5.4	<0.001*	0.304*
HADS Anxiety	6.2	3.9	6.3	3.6	0.698	0.100
Testicle	5.2	3.2	6.7	3.7	0.068	−0.075
Breast	6.7	4.5	5.6	3.6	0.097	0.162
Other	6.6	3.7	6.8	3.8	0.021*	0.142
HADS depression	4.8	3.5	4.4	2.9	0.141	0.150*
Testicle	3.4	2.9	3.9	3.1	0.361	0.169
Breast	5.6	3.9	4.2	2.8	0.021*	0.102
Other	5.2	3.6	4.7	2.9	0.377	0.242

Note

Descriptive data of Avoidance, Intrusion, Anxiety and Depression (IES and HADS scores)

Bold for all cases for test. Italic for subgroups according to the primary tumor.

^a^ Pearson correlation.

^b^ *p* value of the paired *t*‐test.

* *p* < 0.05.

The prevalence of symptoms and disorders across patients and caregivers, and their relative interdependence, are reported in Table 3. On the basis of the defined cut‐off values of the total IES scores, 20.0% of survivors and 35.5% of caregivers had possible PTSD, while 23 patients (11.0%) and 33 caregivers (15.6%) had probable PTSD. Moreover, interdependence, as measured by Cohen's *k*, appears to be significant for mild and severe PTSS (*k* = 0.236 and *k* = 0.161) and depression (*k* = 0.144), but not for anxiety.

**TABLE 3:** PTSS as total IES by category

	Survivors n (%)	Caregivers n (%)	Kappa value
Mild PTSS (IES ≤ 24)	93 (44.1)	68 (32.2)	0.236*
Moderate PTSS (IES: 25‐34)	75 (35.7)	68 (32.2)	0.044
Severe PTSS /possible PTSD (IES ≥ 35)	42 (20.0)	75 (35.5)	0.161*
Depression (HADS‐D ≥ 8)	42 (20.1)	31 (16.1)	0.144*
Anxiety/Arousal (HADS‐A ≥ 8)	62 (29.7)	63 (32.6)	0.097
Probable PTSD (HADS‐A ≥ 8 and IES ≥ 35)	23 (11.0)	33 (15.6)	0.179*

Abbreviations: IES, Impact of Event Scale; PTSS, Post‐Traumatic Stress Symptoms.

* *p* < 0.05.

### 3.3 Pairwise correlations between symptom scores

Among each group of members of the dyad, the severity of avoidance and intrusion were strongly intercorrelated (*R* = 0.68 among survivors and *R* = 0.75 among caregivers), as were anxiety and depression (*R* = 0.72 among survivors and *R* = 0.64 among caregivers), as displayed in Table 4. Among survivors, significant intercorrelations were also detected between all other pairs of symptoms. Whereas among caregivers, depression, but not anxiety, correlated significantly with avoidance or intrusion. The correlations of the same type of symptoms across members of the dyad (e.g. survivor's avoidance and caregiver's avoidance) were weaker, but statistically significant (*R* between 0.15 and 0.29) with the exception of anxiety, which was not significant.

**TABLE 4:** Pearson's correlations between PTSS and depression in patients and caregivers

		Survivor				Caregiver			
		Avoidance	Intrusion	Anxiety	Depression	Avoidance	Intrusion	Anxiety	Depression
Survivor	Avoidance	1							
	Intrusion	0.678	1						
	Anxiety	0.259	0.324	1					
	Depression	0.151	0.212	0.724	1				
Caregiver	Avoidance	0.285	0.175	0.074	0.099	1			
	Intrusion	0.258	0.264	0.151	0.166	0.748	1		
	Anxiety	0.031	0.008	0.100	0.143	0.129	0.127	1	
	Depression	0.023	0.007	0.155	0.150	0.284	0.240	0.640	1

### 3.4 Survivors’ and caregivers’ PTSS

We examined the effects of sociodemographic and clinical variables on the severity of each facet of PTSS, among patients and caregivers separately (Table 5).

**TABLE 5:** Factors associated with survivor and caregiver symptoms

Survivors	PTSS avoidance			PTSS intrusion			PTSS anxiety		
	*R* ^2^ = 12.8%; *F* = 3.939; *p* < 0.001			*R* ^2^ = 22.4%; *F* = 6.783; *p* < 0.001			*R* ^2^ = 68.6%; *F* = 44.654; *p* < 0.001		
	*B*	95% CI	*p*‐value	*B*	95% CI	*p*‐value	*B*	95% CI	*p*‐value
Age	−0.017	−0.087; 0.054	0.644	−0.017	−0.066; 0.033	0.505	−0.030	−0.057; −0.004	0.025
Gender	−1.174	−3.183; 0.822	0.251	−0.266	−1.680; 1.148	0.533	0.972	0.214; 1.730	0.012
Education	−0.250	−1.322; 0.822	0.646	−0.239	−0.993; 0.515	0.533	0.224	−0.180; 0.628	0.276
Treatment	0.626	−1.090; 2.341	0.473	0.673	−0.534; 1.880	0.273	−0.621	−1.268; 0.026	0.060
Previous psychopathology	0.246	−1.577; 2.069	0.790	−1.360	−2.643; −0.077	0.038	0.840	0.152; 1.527	0.017
Relationship^a^	1.228	−0.759; 3.215	0.224	1.331	−0.067; 2.729	0.062	0.524	−0.226; 1.273	0.170
Stressful events	0.116	−1.674; 1.906	0.898	0.876	−0.383; 2.136	0.172	0.501	−0.174; 1.176	0.145
HADS Depression	0.000	−0.302; 0.301	0.998	0.074	−0.139; 0.286	0.496	0.616	0.502; 0.729	<0.001
MiniMAC Anxious Preoccupation	0.554	0.253; 0.856	<0.001	0.512	0.299; 0.724	<0.001	0.165	0.051; 0.279	0.005
MiniMAC Helpless/Hopeless	−0.055	−0.341; 0.231	0.705	−0.089	−0.290; 0.113	0.386	0.102	−0.006; 0.210	0.064

Caregivers	PTSS avoidance			PTSS intrusion			PTSS anxiety		
	*R* ^2^ = 9.9%; *F* = 4.478; *p* < 0.001			*R* ^2^ = 6.9%; *F* = 3.346; *p* = 0.004			*R* ^2^ = 47.1%; *F* = 29.034; *p* < 0.001		
	*B*	95% CI	*p*‐value	*B*	95% CI	*p*‐value	*B*	95% CI	*p*‐value
Age	0.014	−0.068; 0.096	0.734	0.022	−0.043; 0.086	0.510	−0.022	−0.055; 0.011	0.192
Gender	−0.301	−2.404; 1.801	0.778	0.274	−1.370; 1.917	0.743	1.359	0.516; 2.201	0.002
Education	−0.762	−1.886; 0.361	0.182	−0.657	−1.536; 0.222	0.142	0.316	−0.134; 0.766	0.168
Treatment	−0.086	−2.000; 1.829	0.930	0.024	−1.473; 1.520	0.975	−0.444	−1.210; 0.323	0.255
Relationship^a^	−1.563	−3.039; −0.086	0.038	−0.841	−1.996; 0.313	0.152	−0.251	−0.842; 0.340	0.404
HADS depression	0.510	0.168; 0.851	0.004	0.322	0.055; 0.589	0.018	0.848	0.711; 0.985	<0.001

Abbreviations: 95% CI, 95% confidence interval; HADS, Hospital Anxiety and Depression Scale; IES, Impact Of Event Scale; MAC, mental adjustment to cancer; PTSS, post‐traumatic stress symptoms.

Table 5. Caregivers’ PTSS Multivariate Regression Model.

^a^ Spouse or offspring.

Among patients, avoidance symptoms were associated with the tendency of displaying an anxious coping style (*R* ^2^ = 12.8%, *F* = 3.939, *p* < 0.001). The intrusion was negatively associated with previous episodes of psychopathology and with anxious coping style scores (*R* ^2^ = 22.4%; *F* = 6.783; *p* < 0.001). Anxiety was negatively associated with age, female gender, previous episodes of psychopathology, depression and anxious coping style (*R* ^2^ = 68.6%; F = 44.654; *p* < 0.001).

Among caregivers, greater avoidance symptoms were associated with a closer relationship with the patient (being spouse or offspring vs. other relationship) and with higher severity of depression (*R* ^2^ = 9.9%; *F* = 4.478; *p* < 0.001). Intrusion was only associated with higher depression severity (*R* ^2^ = 6.9%; *F* = 3.346; *p* = 0.004); anxiety was associated with both the gender of the caregiver, being it worse when the caregiver is female, and depression severity (*R* ^2^ = 47.1%; *F* = 29.034; *p* < 0.001).

### 3.5 Actor‐Partner Interdependence Models

**FIGURE 1:**
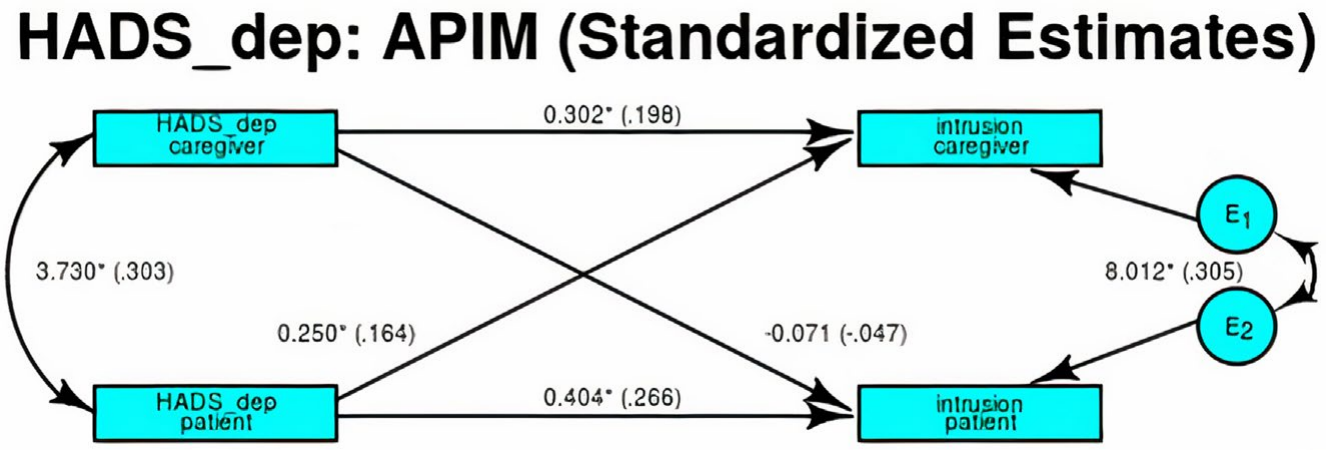
The APIM examining the association between depression and intrusion

We run four Actor‐Partner Interdependence Models (APIM) to examine the association between different facets of PTSD and depression, while accounting for the interdependence of symptoms between members of the dyad. The APIM identified 184 dyads not presenting any missing value. The APIM examining the association between depression and avoidance revealed only significant, small‐sized actor effects (patients standardized effect: 0.199, *p* = 0.006, overall *R* ^2^ = 0.032; caregivers: 0.247, *p* = 0.003, *R* ^2^ = 0.056), while partner effects for both patients and caregivers were not significant. Results of the analysis supported that members of the dyad could be statistically distinguished. The correlation among members ignoring predictors was small (0.338) and explained 1.69% of the total nonindependence. The APIM examining the association between depression and intrusion revealed significant, albeit small‐sized actor effects (patients standardized effect: 0.266, *p* < 0.001, overall *R* ^2^ = 0.069; caregivers: 0.198 *p* = 0.017, *R* ^2^ = 0.064; Figure 1). The partner effects for patients to caregivers was also significant (standardized effect: 0.164, *p* = 0.040), while that of caregivers to patients was not. Results of the analysis supported that members of the dyad could be statistically distinguished. The correlation between members ignoring predictors was small (0.332) and the model explained 15.2% of the total nonindependence. The APIM examining the association between depression and anxiety revealed significant, large actor effects (patients standardized effect: 0.693, *p* < 0.001, overall *R* ^2^ = 0.494; caregivers: 0.569, *p* < 0.001, *R* ^2^ = 0.298). Whereas, the partner effects were not significant. Results supported that members of the dyad could be statistically distinguished. The correlation between members ignoring predictors was small (0.106) and the model explained 77.9% of the total nonindependence. The APIM examining the association among intrusion, anxiety and avoidance symptoms explained a consistent part of the variance of avoidance (patients *R* ^2^ = 0.464; caregivers *R* ^2^ = 0.555). The model revealed that intrusion had significant, large actor effects (patients standardized effect: 0.658, *p* < 0.001; caregivers: 0.753, *p* < 0.001), but non‐significant partner effects. Whereas, anxiety did not have a significant actor or partner effects. Results supported that members of the dyad could not be statistically distinguished. The correlation between members ignoring predictors was small (0.286) and the model explained 62.80% of the total nonindependence.

## 4 DISCUSSION

This study examined the correlates and reciprocal associations of post‐traumatic symptoms among adult cancer survivors and their caregivers. The main findings were: (1) nearly 20% of patients and, even more strikingly, up to 30% of caregivers reported severe post‐traumatic symptoms 5 years or more since the diagnosis of cancer; (2) among cancer patients the severity of post‐traumatic symptoms was associated with an anxious coping style, previous psychopathology and depression, whereas among caregivers it was associated with depression and having a closer relationship with patients, in particular, caregivers who were spouses or siblings experienced significantly more avoidance symptoms than other caregivers; (3) taking in account dyadic symptom interactions, patients’ depression was associated with caregivers’ intrusion symptoms. This study is the first, to our knowledge, to address the relationship between cancer patients and caregivers post‐traumatic symptoms, especially using a dyadic method.

Post‐traumatic stress disorder is a common, debilitating occurrence among cancer patients and may become evident since the early stages of cancer management.^16^, ^17^, ^18^ This study, together with few others,^10^, ^12^ shows that high levels of cancer‐related PTSS may persist even several years after treatment among both patients and caregivers. Unlike previous investigations, we examined caregivers of adult patients, thus extending previous findings: nearly one out of five survivors, and one out of three caregivers, experienced severe PTSS. Perhaps even more surprising is that caregivers displayed slightly higher severity of PTSS than survivors, irrespective of the operational criteria. These findings are consistent with those of Mehnert and colleagues,^10^ who reported a prevalence of 12% PTSD among cancer survivors, and those of Shelby et al. who found that 10%–20% of breast cancer survivors presented with subsyndromal PTSD.^12^ These figures are consistent with those of the only other study, to our knowledge, that examined caregivers of adult cancer long‐term survivors. Here, high rates of both full‐fledged PTSD and subthreshold post‐traumatic symptoms were detected between caregivers and patients.^23^ These results stress the importance of actively continue to evaluate post‐traumatic symptoms among oncologic patients years after the diagnosis, similarly to what is currently advised among individuals who experienced other life‐threatening conditions.^46^, ^47^

Previous literature on post‐traumatic disorders suggests that individuals who are indirectly exposed to traumatic events can develop PTSD, although with a lower probability than those directly involved.^48^ Our study shows that in the context of cancer, caregivers may be similar or even more affected than their counterparts.^49^ Furthermore, caregivers may also experience other symptoms of psychological distress, such as anxiety and depression, with similar or greater severity than patients.^50^, ^51^ We also observed an association between depression and post‐traumatic symptoms’ dimensions among caregivers, as well as between depression *in patients* and intrusion symptoms *in caregivers*.^52^, ^53^ While these associations should not be interpreted as necessarily causal, they reinforce the notion of interdependence between cancer patients’ and caregivers’ mental health, in line with dynamic models of psychopathology.^54^, ^55^ These findings reinforce the need for a dimensional, interpersonal understanding of psychopathology, as literature on PTSD has already pointed out.^54^ In this view, caregivers’ intrusion symptoms could be interpreted as an effect of indirect traumatization, which seems particularly relevant among those patients who develop persistent depression.^56^ Interactions between patients’ and caregivers PTSS may show similarities with ‘relational PTSD’, a disorder which was mainly investigated in pediatric oncology and is seemingly due to child‐parent mutual influences of psychological stress.^57^, ^58^ Adopting such a symptom dynamic perspective, it might be hypothesized that features of depression displayed by the patient (e.g. ruminations, interpersonal withdrawal) might act as emotional cues triggering or reinforcing caregivers’ intrusion memories. Further studies, however, are warranted to clarify the psychological dynamics among adult cancer survivors and their caregivers.

Long‐term PTSD and post‐traumatic symptoms have been almost neglected by literature, but they clearly deserve clinical attention, in cancer patients and their caregivers. The findings of this study suggest to assess (screening or monitoring) cancer‐related PTSD and PTSS even in the long term, given their potential impact on quality of life, disability and risk of further psychiatric and medical morbidity.^59^ This is especially relevant considering that several safe and effective treatments are available, including psychotherapy.^59^

This study is among the few that examined post‐traumatic symptoms in cancer survivors and their caregivers, years after the diagnosis of cancer. Its strengths include a large sample size, concurrent investigation of PTSS and depression, as well as analyses that account for dyadic interactions. However, limitations must be considered: first, the design is cross‐sectional and does not allow direct causal inferences.^14^ Second, results obtained using screening instruments should be replicated with studies employing structured psychiatric interviews, especially in the light of recent changes in DSM‐5 criteria for PTSD. Third, participants were recruited from a single comprehensive cancer centre and this could limit the generalizability of results, especially for cases that did not indicate family caregivers (24%). Fourth, the assessment of PTSD was not based on the revised version of the IES, which also comprises a measure of the arousal. Fifth, only a minority of patients displayed a metastatic disease and/or a relapse, not allowing for a thorough appreciation of their influence on PTSS; further studies with larger samples will be able to examine this issue more in depth.

### 4.1 Conclusions

In conclusion, long‐term cancer survivors and their caregivers are affected by significant levels of post‐traumatic symptoms, which seem to outlast successful treatment by several years. Post‐traumatic psychopathology may be particularly affected by depression, especially in the context of interpersonal dynamics. These findings highlight the need to maintain high levels of clinical vigilance among cancer survivors as well as their caregivers, taking into account the dyadic psychological morbidity and distress.^60^ Future research might need to take into account such complex between‐person psychopathology dynamics.

## CONFLICT OF INTEREST

The authors declare that there is no conflict of interest.

## AUTHOR CONTRIBUTIONS

**Silvia De Padova** designed and coordinated the study, was involved in data collection, interpreted results, and drafted the manuscript. **Luigi Grassi** co‐designed the study, interpreted results, and co‐drafted the manuscript. **Lorena Rossi, Alberto Farolfi, Alessandro Passardi, Tatiana Bertelli,** and **Alejandra Berardi** were involved in data collection and interpretation. **Alessandro Vagheggini,** **Martino Belvederi Murri,** and Federica Folesani performed the statistical analysis and extensive reviews of the manuscript. **Ugo De Giorgi** co‐designed and ‐coordinated the study, interpreted results, and co‐drafted the manuscript. All authors read and approved the final version of the manuscript for submission.

## TABLE OF CONTENTS

- High levels of cancer‐related post‐traumatic stress symptoms (PTSS) exist for many years after treatment in both long‐term cancer survivors and their caregivers.
- Prospective studies are warranted to identify PTSS and implement appropriate interventions.

## Supporting information

Table S1

Supplementary Material

## Data Availability

The datasets generated and/or analysed during the current study are available from the corresponding author on reasonable request.
