# Supplementary material for: Post‐traumatic stress symptoms in long‐term disease‐free cancer survivors and their family caregivers
Source: Cancer Med. 2021 Jun 1;10(12):3974–85. doi: 10.1002/cam4.3961 (PMC8209622; doi:10.1002/cam4.3961)
Supplement: Supplementary file 1 — Table S1 [file CAM4-10-3974-s002.doc]

Supplementary Table 1. HADS anxiety and depression scores by categories.

|  | **Survivors**  **n (%)** | **Caregivers**  **n (%)** | ***P-*value**^1^ |
| --- | --- | --- | --- |
| *HADS anxiety* |  |  |  |
| Probable case (≥ 11) | 28 (13.3) | 31 (14.7) | 0.587 |
| Borderline | 33 (15.7) | 37 (17.5) |
| Non-case (≤ 7) | 149 (71.0) | 143 (67.8) |
| Missing | 2 | 1 |  |
| *HADS depression* |  |  |  |
| Probable case (≥ 11) | 17 (8.1) | 5 (2.4) | 0.001 |
| Borderline | 24 (11.4) | 31 (17.7) |
| Non-case (≤ 7) | 169 (80.5) | 175 (82.9) |
| Missing | 2 | 1 |  |

Abbreviations: HADS, Hospital Anxiety and Depression Scale.

^1^chi-squared test for association.
