# Supplementary material for: Post‐traumatic stress symptoms in long‐term disease‐free cancer survivors and their family caregivers
Source: Cancer Med. 2021 Jun 1;10(12):3974–85. doi: 10.1002/cam4.3961 (PMC8209622; doi:10.1002/cam4.3961)
Supplement: Supplementary file 2 — Supplementary Material [file CAM4-10-3974-s001.docx]

**Output of APIM models**

**1. Association between depression and avoidance symptoms**


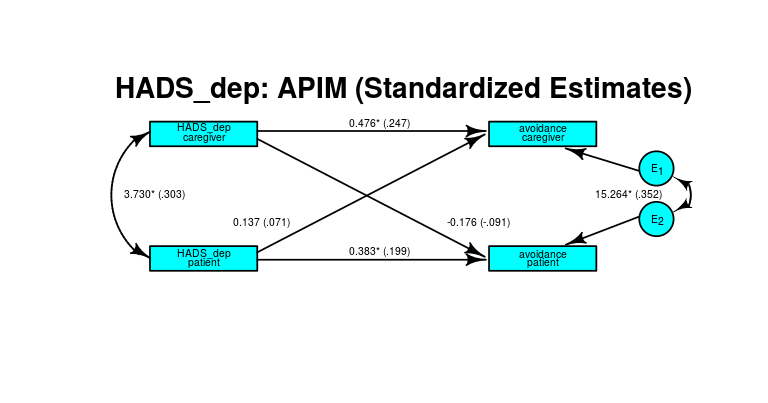


Table 1: Descriptive Statistics

Variable Role Mean SD Minimum Maximum
avoidance patients 18.344 6.359 7.000 34.000
 caregivers 20.739 7.144 10.000 40.000
 HADS_dep patients 4.939 3.569 0.000 19.000
 caregivers 4.578 3.449 0.000 21.000

Table 2: Separate Effect Estimates for the Actor-Partner Interdependence Model for patients and caregivers

Variable Role Effect Estimate Lower 95.00 CI Upper p value Beta (o) Beta (s) r
avoidance patients Intercept 17.261 15.471 to <.001 <.001
 caregivers 17.883 15.897 to 19.869 <.001
 HADS_dep patients Actor 0.383 0.113 to 0.652 .006 0.199 0.215 .205
 Partner -0.176 -0.455 to 0.103 .216 -0.091 -0.096 -.093
 k -0.460 -1.569 to 0.397
 caregivers Actor 0.476 0.167 to 0.785 .003 0.247 0.230 .221
 Partner 0.137 -0.162 to 0.436 .369 0.071 0.068 .067
 k 0.288 -0.316 to 1.691

Table 3: Overall Effect Estimates for the Actor-Partner Interdependence Model

Variable Effect Estimate Lower 95.00 CI Upper p value Beta r
avoidance Intercept 17.572 16.019 to 19.126 <.001
 HADS_dep Actor 0.429 0.235 to 0.623 <.001 0.220 .213
 Partner -0.020 -0.213 to 0.174 .843 -0.010 -.010
 k -0.046 -0.580 to 0.433

Table 4: Tests of Distinguishability
 Eq. Ints. Eq Actor Eq. Partner Eq. Error Vars. chi square df p
 Yes Yes Yes Yes 26.016 4 <.001
 Yes No No No 0.325 1 .569
 No Yes Yes No 4.527 2 .104
 No Yes No No 0.183 1 .669
 No No Yes No 2.065 1 .151
 No No No Yes 2.158 1 .142
 No Yes Yes Yes 6.713 3 .082

Table 5: Partition of Nonindependence
 Source of Correlation Amount % Total Amount % Total
 Overall Correlation .338 100.00
 Total Due the APIM .006 1.69
 Spurious Due to A&P Effects -.007 -2.15
Correlation of the A&P Variables .013 3.84
 Unexplained Correlation .332 98.31

**2. Association between depression and intrusion symptoms**


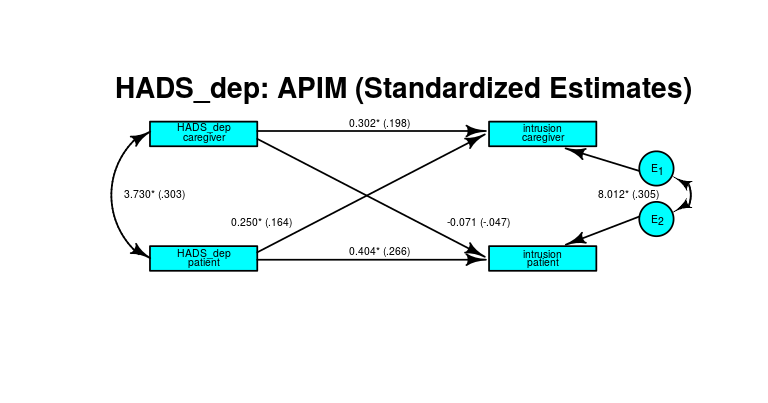


Table 1: Descriptive Statistics

Variable Role Mean SD Minimum Maximum
intrusion patients 11.928 4.929 2.000 29.000
 caregivers 14.833 5.715 1.000 28.000
 HADS_dep patients 4.939 3.569 0.000 19.000
 caregivers 4.578 3.449 0.000 21.000

Table 2: Separate Effect Estimates for the Actor-Partner Interdependence Model for patients and caregivers

Variable Role Effect Estimate Lower 95.00 CI Upper p value Beta (o) Beta (s) r
intrusion patients Intercept 10.260 8.899 to <.001 <.001
 caregivers 12.218 10.636 to 13.800 <.001
 HADS_dep patients Actor 0.404 0.199 to 0.609 <.001 0.266 0.292 .279
 Partner -0.071 -0.283 to 0.141 .510 -0.047 -0.050 -.050
 k -0.177 -0.713 to 0.458
 caregivers Actor 0.302 0.055 to 0.548 .017 0.198 0.182 .178
 Partner 0.250 0.012 to 0.488 .040 0.164 0.156 .153
 k 0.828 -0.027 to 4.608

Table 3: Overall Effect Estimates for the Actor-Partner Interdependence Model

Variable Effect Estimate Lower 95.00 CI Upper p value Beta r
intrusion Intercept 11.239 10.049 to 12.430 <.001
 HADS_dep Actor 0.353 0.200 to 0.506 <.001 0.224 .224
 Partner 0.089 -0.063 to 0.241 .250 0.057 .058
 k 0.253 -0.182 to 0.824

Table 4: Tests of Distinguishability

Eq. Ints. Eq Actor Eq. Partner Eq. Error Vars. chi square df p
 Yes Yes Yes Yes 47.325 4 <.001
 Yes No No No 4.854 1 .028
 No Yes Yes No 3.907 2 .142
 No Yes No No 0.364 1 .546
 No No Yes No 3.611 1 .057
 No No No Yes 4.398 1 .036
 No Yes Yes Yes 8.359 3 .039

Table 5: Partition of Nonindependence

 Source of Correlation Amount % Total Amount % Total
 Overall Correlation .332 100.00
 Total Due the APIM .050 15.17
 Spurious Due to A&P Effects .037 11.02
Correlation of the A&P Variables .014 4.15
 Unexplained Correlation .281 84.83

**3. Association between depression and anxiety/ arousal symptoms**


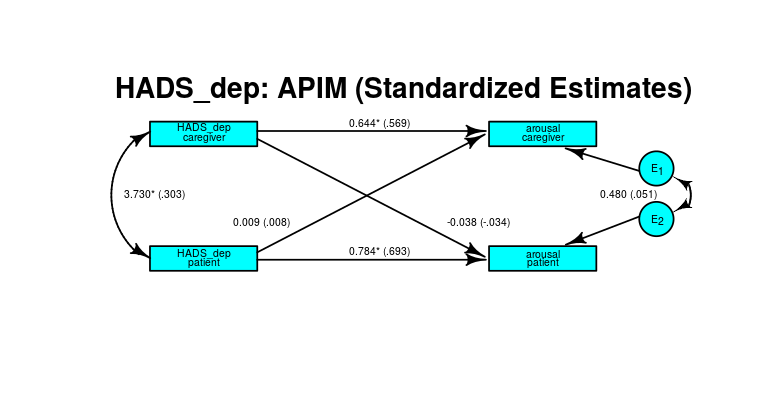


Table 1: Descriptive Statistics

Variable Role Mean SD Minimum Maximum
 arousal patients 6.044 3.906 0.000 21.000
 caregivers 6.272 4.030 0.000 27.000
HADS_dep patients 4.939 3.569 0.000 19.000
 caregivers 4.578 3.449 0.000 21.000

Table 2: Separate Effect Estimates for the Actor-Partner Interdependence Model for patients and caregivers

Variable Role Effect Estimate Lower 95.00 CI Upper p value Beta (o) Beta (s) r
 arousal patients Intercept 2.349 1.554 to <.001 <.001
 caregivers 3.282 2.315 to 4.248 <.001
HADS_dep patients Actor 0.784 0.664 to 0.903 <.001 0.693 0.716 .694
 Partner -0.038 -0.162 to 0.086 .545 -0.034 -0.034 -.046
 k -0.049 -0.200 to 0.116
 caregivers Actor 0.644 0.493 to 0.794 <.001 0.569 0.551 .533
 Partner 0.009 -0.136 to 0.154 .904 0.008 0.008 .009
 k 0.014 -0.201 to 0.266

Table 3: Overall Effect Estimates for the Actor-Partner Interdependence Model

Variable Effect Estimate Lower 95.00 CI Upper p value Beta r
 arousal Intercept 2.816 2.174 to 3.457 <.001
HADS_dep Actor 0.714 0.618 to 0.809 <.001 0.632 .613
 Partner -0.015 -0.109 to 0.080 .762 -0.013 -.016
 k -0.021 -0.150 to 0.116

Table 4: Tests of Distinguishability

Eq. Ints. Eq Actor Eq. Partner Eq. Error Vars. chi square df p
 Yes Yes Yes Yes 11.210 4 .024
 Yes No No No 2.268 1 .132
 No Yes Yes No 2.038 2 .361
 No Yes No No 2.038 1 .153
 No No Yes No 0.236 1 .627
 No No No Yes 6.655 1 .010
 No Yes Yes Yes 8.819 3 .032

Table 5: Partition of Nonindependence

 Source of Correlation Amount % Total Amount % Total
 Overall Correlation .137 100.00
 Total Due the APIM .106 77.93
 Spurious Due to A&P Effects -.013 -9.47
Correlation of the A&P Variables .119 87.40
 Unexplained Correlation .030 22.07

**4. Association between intrusion, anxiety (predictors) and avoidance symptoms (dependent variable)**


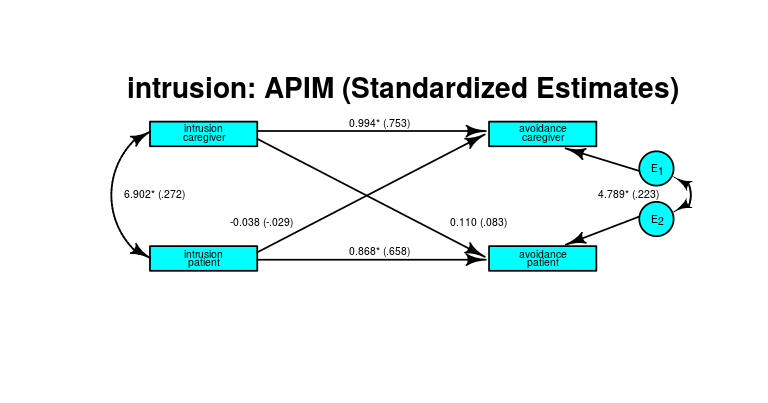


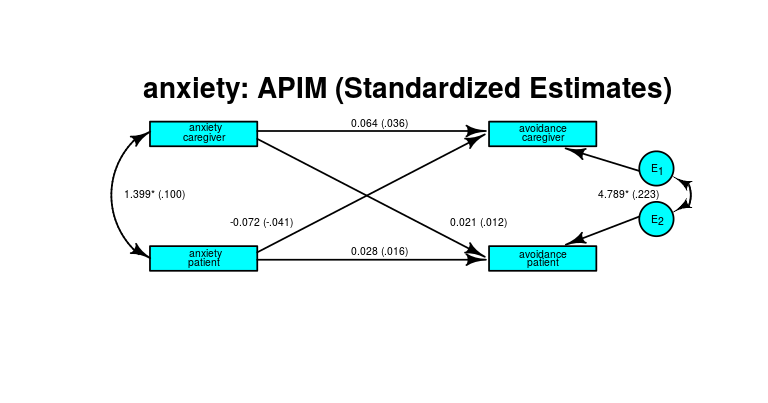


Table 1: Descriptive Statistics

Variable Role Mean SD Minimum Maximum
avoidance patients 18.349 6.359 10.000 34.000
 caregivers 20.615 6.930 10.000 40.000
intrusion patients 11.932 4.777 7.000 28.000
 caregivers 14.802 5.292 6.000 28.000
 anxiety patients 6.161 3.856 0.000 21.000
 caregivers 6.302 3.612 0.000 19.000

Table 2: Separate Effect Estimates for the Actor-Partner Interdependence Model for patients and caregivers

Variable Role Effect Estimate Lower 95.00 CI Upper p value Beta (o) Beta (s) r
avoidance patients Intercept 6.054 3.555 to <.001 <.001
 caregivers 6.404 3.921 to 8.886 <.001
intrusion patients Actor 0.868 0.716 to 1.020 <.001 0.658 0.652 .633
 Partner 0.110 -0.021 to 0.241 .101 0.083 0.092 .119
 k 0.127 -0.023 to 0.297
 caregivers Actor 0.994 0.863 to 1.124 <.001 0.753 0.759 .738
 Partner -0.038 -0.189 to 0.113 .619 -0.029 -0.026 -.036
 k -0.039 -0.186 to 0.117
 anxiety patients Actor 0.028 -0.156 to 0.213 .763 0.016 0.017 .022
 Partner 0.021 -0.164 to 0.206 .826 0.012 0.012 .016
 k 0.732 -11.479 to 12.103
 caregivers Actor 0.064 -0.120 to 0.248 .494 0.036 0.034 .050
 Partner -0.072 -0.256 to 0.111 .440 -0.041 -0.040 -.056
 k -1.127 -12.654 to 12.147

Table 3: Overall Effect Estimates for the Actor-Partner Interdependence Model

Variable Effect Estimate Lower 95.00 CI Upper p value Beta r
avoidance Intercept 6.229 4.281 to 8.176 <.001
intrusion Actor 0.931 0.833 to 1.029 <.001 0.723 .686
 Partner 0.036 -0.062 to 0.133 .471 0.028 .036
 k 0.038 -0.067 to 0.144
 anxiety Actor 0.046 -0.083 to 0.175 .482 0.026 .036
 Partner -0.026 -0.155 to 0.103 .695 -0.014 -.020
 k -0.557 -10.203 to 10.716

Table 4: Tests of Distinguishability

Eq. Ints. Eq Actor Eq. Partner Eq. Error Vars. chi square df p
 Yes Yes Yes Yes 3.997 6 .677
 Yes No No No 0.050 1 .823
 No Yes Yes No 3.532 4 .473
 No Yes No No 1.780 2 .411
 No No Yes No 3.153 2 .207
 No No No Yes 0.009 1 .926
 No Yes Yes Yes 3.541 5 .617

Table 5: Partition of Nonindependence

 Source of Correlation Amount % Total Amount % Total
 Overall Correlation .286 100.00
 Total Due the APIM .180 62.80
 Spurious Due to A&P Effects .052 18.16
 Correlation of the A&P Variables .134 46.77
Correlation between the Mixed Variables -.006 -2.12
 Unexplained Correlation .106 37.20
